# Associations of physical activity and sedentary behaviors with child mental well-being during the COVID-19 pandemic

**DOI:** 10.1186/s12889-021-11805-6

**Published:** 2021-09-28

**Authors:** Amanda S. Gilbert, Laurel Schmidt, Alan Beck, Maura M. Kepper, Stephanie Mazzucca, Amy Eyler

**Affiliations:** Prevention Research Center in St. Louis, Brown School at Washington University in St. Louis, St. Louis, MO USA

**Keywords:** Mixed-methods, Child, Mental well-being, Physical activity, Sedentary behavior, COVID-19

## Abstract

**Background:**

The COVID-19 pandemic resulted in public health and policy measures to reduce in-person contact and the transmission of the virus. These measures impacted daily life and mental well-being (MWB). The aims of this study were to explore the MWB impacts of COVID-19 on children and assess the associations among perceived changes in physical activity (PA) and sedentary behaviors (SB), with perceived MWB changes, using a mixed-methods approach.

**Methods:**

A convergent parallel mixed-methods design consisting of an online survey with a convenience sample and interviews was conducted from May through July 2020 with parents/caregivers of kindergarten through 5th graders in the St. Louis region. Survey domains assessed included child MWB, PA, and SB. Interviews were recorded, transcribed, and qualitatively analyzed using a code book developed to elicit themes. Survey data was analyzed with chi-squared tests and logistic regressions. The dependent variable was perceived change in child MWB due to the impact of COVID-19. Independent variables included perceived changes in PA, SB, and child concerns about COVID-19.

**Results:**

Sample size consisted of 144 surveys and 16 interviews. Most parents reported a perceived decrease in child MWB (74%), a decrease in child PA (61%), and an increase in child SB (91%). Discontentment with stay-at-home orders and concern about COVID-19 were associated with a perceived decrease in MWB. Children whose PA decreased were 53% less likely to have the same or better MWB (OR 0.47) and children whose outside PA decreased were 72% less likely to have the same or better MWB (OR 0.28). Common qualitative themes included difficulty in adjusting to COVID-19 restrictions due to school closures and lack of socializing, child concerns about family getting sick, and PA benefits for improving MWB.

**Conclusions:**

Based on parent perceptions, MWB decreased with COVID-19. Maintained or increased child PA improved the chances MWB would remain the same or improve. Parent interviews provide context to these findings by showing how COVID-19 impacted MWB and the associations between PA and MWB. Understanding protective factors for child MWB during COVID-19 is important to offset negative long-term health outcomes from this ongoing pandemic.

**Supplementary Information:**

The online version contains supplementary material available at 10.1186/s12889-021-11805-6.

## Background

Beginning in December 2019, the respiratory disease SARS-CoV-2 virus (COVID-19) began spreading around the world. Since then, the World Health Organization has reported over 200 million cases and over 4 million deaths worldwide, with the Centers for Disease Control and Prevention reporting over 36.7 million cases and around 620,000 deaths in the United States (US) as of August 15, 2021 [1, 2]. To prevent COVID-19 transmission in the US, public health measures such as stay-at-home orders, closures of non-essential businesses, in-person school, and many other public places have been implemented to restrict in-person contact. These restrictions are ongoing and have had a significant impact on daily life and well-being [3–5]. Preliminary research in the first few months of the COVID-19 pandemic identified child mental well-being (MWB) as being particularly vulnerable to the pandemic and associated public health restrictions [6–11]. Children face social isolation, educational delays from in-person school closures, and stress from financial strains many families experience because of parents and caregivers losing work due to COVID-19 restrictions [7, 10, 12, 13]. Additionally, children of elementary school age may have a hard time understanding the health risks of COVID-19 and changes to daily life from lockdown measures, further increasing fear and anxiety [6]. COVID-19 restrictions are also associated with increased sedentary behavior (SB) and decreased physical activity (PA), which further increase the risk for poor mental health and well-being in children [14–20].

The full impacts of COVID-19 on MWB, PA, and SB are still unknown; however, evidence suggests poor MWB, limited PA, and increased SB in childhood may have potentially long-term and serious health consequences [21–26]. Children who do not meet recommended PA guidelines (i.e., 60 min of moderate-vigorous PA per day) are at an increased risk of developing mental health disorders, such as depression and anxiety, as well as chronic diseases, including obesity, diabetes, and cardiovascular disease later in life [22, 27]. Children not only need regular PA to improve health and well-being, but can be vulnerable to decreased cognitive functioning, obesity, and cardiometabolic risk factors if they spend significant amounts of time being sedentary (e.g. sitting, watching TV, on the computer) [27–29].

The COVID-19 pandemic and associated restrictions are ongoing with no clear timeframe for when restrictions will be lifted, making the potential impacts on child MWB, PA, and SB with the subsequent long-term effects on health and well-being particularly concerning. Given this, the aims of this study were 1) to explore how COVID-19 restrictions are impacting child MWB, PA, and SB using qualitative methods, and 2) to assess how perceived changes in PA and SB due to COVID-19 restrictions are associated with perceived changes in MWB using quantitative methods.

## Methods

### Study design

This was a convergent parallel mixed-methods study to assess the impact of COVID-19 on child MWB and associations with PA and SB. Convergent parallel designs consist of conducting qualitative and quantitative methods independently and concurrently, with results from each of these methods being integrated during the interpretation of findings [30]. The purpose of using this design was to triangulate results from each method, and to further understand the context of quantitative results through qualitative findings. Online surveys were conducted with parents and caregivers of children in kindergarten through 5th grade to assess the association among perceived changes in PA and SB, and perceived MWB changes. Surveys were conducted concurrently with the same population to explore the MWB impacts of COVID-19 on children. Following analysis, results from the online survey and interviews were integrated to inform study interpretations.

### Recruitment and participants

Participants for the online survey were recruited using convenience sampling. The survey link was posted on Twitter and Facebook and distributed on Facebook to St. Louis-based neighborhood groups for parents with elementary school children. Sampling methods focused on parents and caregivers in the St. Louis region with children enrolled in kindergarten through 5th grade. The survey was available from late May to early July 2020 for approximately 8 weeks. Participants had the option of providing email addresses. Interviews were conducted independently during this time period within the same population, using a combination of convenience and snowball sampling. Participants were contacted via phone and email. The Institution Review Board of Washington University in St. Louis approved this study with an exempt status.

### Procedures

Participants completed the online survey via Qualtrics. All surveys were anonymous and consisted of a consent page and information regarding the purpose of the study. The online survey was developed for this study (see Supplement 1). The survey consisted of 63 questions and took between 15 and 20 minutes to complete. Topics in the survey included parent, child, and household demographics, child MWB, child PA and SB, as well as parental influence and rules regarding these behaviors. Qualitative interviews (*n* = 16) were administered via telephone or Zoom and lasted between 20 and 30 min. Parents were provided the consent form and reason for this study prior to the interview. Participants completed the interviews from their homes in privacy. No repeat interviews were conducted. Interviews were conducted until saturation was reached. Interviews were audio recorded for accuracy, de-identified, and then professionally transcribed verbatim. If participants had more than one child, participants were asked to choose one child to answer questions for. Questions asked during the interviews were informed by early COVID-19 research and developed with expertise of the research team. Questions covered similar topics to the online survey, along with more open-ended explorations around how the COVID-19 pandemic impacted their families and children.

### Measures

#### Perceived changes in child MWB

##### Quantitative

An adapted version of the parent-report on child Mood and Feelings Questionnaire was used to measure child MWB [31]. Items in the measure were chosen based on face value relevance for measuring COVID-19 impact and stems were modified to focus on the time period prior to and during COVID-19 restrictions. Parents were asked to answer whether statements were true, sometimes true, or not true about their child. The questionnaire consisted of 9 statements regarding whether the child was miserable or unhappy, not enjoying anything at all, tired, very restless, finding it hard to think properly and concentrate, lonely, irritable or angry, worried a lot, and having trouble sleeping. Parents filled out this questionnaire based on perceptions of how their child felt prior to and during COVID-19 restrictions. Scores were given to each answer (true = 2, sometimes true = 1, not true = 0), and added up to create MWB scores for before and during COVID-19 restrictions. Higher scores indicated worse MWB. To get changes in MWB, the total MWB score for each child during COVID-19 restrictions was subtracted from the total MWB score prior to COVID-19 restrictions and then dichotomized into same or better MWB and worse MWB.

##### Qualitative

Interviews assessed child MWB by asking parents about their child’s general mood and how their child’s mood had been impacted by COVID-19 restrictions. Questions were meant to elicit qualitative responses on how parents perceived their child’s MWB and to explore context around why or how MWB might be impacted by COVID-19 restrictions. Follow up questions were often asked about the impact of school closures, and what parents found to be helpful for improving MWB during COVID-19 restrictions. If parents noted worsening MWB, follow up questions were asked to elicit more information about why these changes to MWB occurred.

#### COVID-19 concerns

##### Quantitative

The online survey assessed COVID-19 concerns by asking parents to report on whether statements such as, my child has not been content with stay-at-home orders, was afraid of self/family/friends getting sick, was concerned about COVID-19, and talked/asked questions about COVID-19, were true, sometimes true, or not true.

##### Qualitative

In interviews with parents, COVID-19 concerns were explored by asking parents about their child’s level of concern regarding COVID-19, their understanding, and what questions and concerns their child voiced about COVID-19.

#### Perceived changes in PA and SB

##### Quantitative

To measure parent perceptions of changes in child PA and SB, questions from an existing scale, the HomeSTEAD’s physical activity and screen media practices and beliefs survey were used [32]. Parents were asked in the online survey whether their child’s PA increased, stayed the same, or decreased due to COVID-19 restrictions. Specific PA behaviors and SB were measured by asking parents to report on the daily number of minutes for activities during COVID-19 restrictions and whether that amount of time was less, more, or about the same as it was prior to the COVID-19 pandemic. PA behaviors included playing inside, playing outside, participating in sports and organized activities, and playing outdoors as a family. SB included using screens (e.g. TV, tablet, computer, smartphone, video games) for entertainment, using screens for educational purposes, and time spent sitting.

##### Qualitative

During interviews parents were asked to describe their child’s current level of PA, and if their child’s PA level had changed due to COVID-19 restrictions. If parents perceived changes to child PA, follow up questions were asked about how COVID-19 restrictions impacted PA levels. Parents were also asked to describe the types of activities their child engaged in during COVID-19 restrictions and how these activities differed from before COVID-19 restrictions. Parents were also asked about SB and how much their child spent time using screens for school and entertainment during COVID-19 restrictions. Follow up questions assessed whether this was different prior to COVID-19 and if so, what the changes had been. Similar questions were asked about non-screen time sedentary activities (e.g. reading, coloring, building blocks).

### Analysis

Quantitative data analysis was conducted using SPSS (version 26). The dependent variable was perceived change in MWB. Independent variables included in the analysis were PA behaviors (perceived change in PA, perceived change in organized sports, perceived change in outside play, perceived change in inside play, and perceived change in family PA), SB (perceived change in time sitting, perceived change in screen time for entertainment, and perceived change in screen time for education), and COVID-19 concerns (discontent with stay-at-home orders, concern about COVID-19, and fear of family/self/friends getting sick). PA and SB were dichotomized into binary variables of ‘same or increased’ and ‘decreased’. COVID-19 concerns were also dichotomized into ‘sometimes true or true’ and ‘not true’. Descriptive statistics, frequency tables, and chi-square analyses were conducted. The McNemar test was run to determine if there were statistically significant differences between each MWB item of the Mood and Feelings questionnaire reported for prior to COVID-19 restrictions compared to during COVID-19 restrictions. For statistically significant correlations, logistic regressions were conducted to determine the association between PA, SB, and COVID-19 concerns with perceived change in MWB. Adjusted models were also run controlling for age and gender.

Qualitative analysis of the transcripts began with three rounds of preliminary analysis to develop the qualitative code book. In the first round, we used an open coding method, were a priori codes were developed based on categories of interview questions from the interview guide. In the second round, two research team members reviewed all transcripts and collaborated to add more detailed codes to existing categories. Additional categories for codes were added as needed. In the third round, two research team members used this initial codebook to double-code three transcripts. The two team members then reviewed the coding to identify any gaps in the codebook or differences in coding. Once consensus on coding was established, the codebook was finalized, and a third research team member was trained to use the final codebook. Using the final codebook, each transcript was double-coded by the three members of the research team and reviewed to eliminate discrepancies between reviewers. Coded text was separated out by code to be reviewed for common themes by research team members. Common themes were discussed and agreed upon through consensus. These themes were then compared to the quantitative findings and integrated by identifying convergence and places where qualitative rich text could be used to provide context for quantitative results.

## Results

The total number of parents surveyed was 245. The final sample consists of the 144 respondents who completed the child Mood and Feelings Questionnaire. The final analytic sample was not significantly different than the total sample of parents surveyed for demographic variables of child age, race, gender, and ethnicity. Table 1 lists the descriptive statistics for the demographic variables of the sample. The ages of children in the quantitative sample ranged from five to twelve years old, with an average age of 8 (SD = 1.75). A majority of children (96%) were white, 44% were female, and most (74%) lived in a household with two to three children. Most of the parents/caregivers surveyed were female (88%), ranging in age from 30 to 51 years old with an average parental age of 40 (SD = 4.17). The majority were white (96%), married or living with a partner (93%), and employed (83%).

**Table 1:** Baseline characteristics of child mental well-being survey sample

**Quantitative survey** (*n* = 144)	
Child characteristics	
Age, years M (SD)	8.01 (1.75)
Female, n (%)	64 (44.44)
White, n (%)	138 (95.83)
Children in household, n (%)	
One	21 (14.58)
Two-Three	106 (73.61)
Four or more	17 (11.81)
Parent characteristics	
Age, years M (SD)	39.76 (4.17)
Female, n (%)	126 (87.50)
White, n (%)	138 (95.80)
Hispanic, Latino, or Spanish origin n (%)	9 (6.30)
Married, living with partner, n (%)	134 (93.10)
Employed, n (%)	120 (83.33)
Employment Location, n (%)	
Outside of home	22 (16.20)
At home	95 (69.90)
Both at & outside of home	19 (14.00)
**Qualitative interviews** (*n =* 16)	
Child characteristics	
Age, years M (SD)	7 (0.71)
Female, n (%)	13 (56.50)^a^
Households with more than one child, n (%)	14 (87.50)
Parent characteristics	
Female, n (%)	15 (93.75)
Married, n (%)	13 (81.25)

^a^Parents reported on a total of 23 elementary school aged children

Sixteen qualitative interviews were conducted with parents who had children ranging in age from four to 11 years old, with an average age of 7 (SD = 0.71). Parents reported on a total of 23 elementary school aged children, 13 of which were female. Overall, 87% of households had more than one child. Parents interviewed were mostly female (94%) and married (81%). Parents reported a wide range of jobs from social worker and teacher, to IT consultant, attorney, and business owner. Most reported working from home and a few noted being laid off or furloughed due to the COVID-19 pandemic.

### Quantitative results

Overall, 74% of parents reported their children had worse MWB during COVID-19 restrictions (see Table 2). The majority of parents (75%) reported their child was discontent with stay-at-home orders. Most parents (76%) reported their child was concerned about COVID-19 and 63% were afraid for themselves or a family member getting sick. Each MWB item in the adapted Mood and Feelings questionnaire was reported to be worse during COVID-19 restrictions. Among aspects of child MWB, perceived loneliness increased the most, from less than 1% prior to COVID-19 restrictions to 33% during COVID-19 restrictions. Other aspects of child MWB perceived to worsen the most were anger, difficulty concentrating, restlessness, difficulty sleeping, and unhappiness.

**Table 2:** Frequencies of parent reported child MWB, PA, and SB before and during COVID- 19 restrictions (*n* = 144)

	Pre- COVID-19	During COVID-19	*P*-value^a^
**Mental well-being, n (%)**^**b**^			
Lonely	1 (0.69)	48 (33.33)	< 0.001
Unhappy	1 (0.69)	16 (11.11)	< 0.001
Lack enjoyment	1 (0.69)	9 (6.25)	< 0.01
Tired	0 (0.00)	10 (6.94)	< 0.001
Restless	7 (4.86)	27 (18.75)	< 0.001
Difficulty concentrating	9 (6.25)	31 (21.52)	< 0.001
Difficulty sleeping	2 (1.39)	20 (13.88)	< 0.001
Worried	6 (4.17)	25 (17.36)	< 0.001
Angry	4 (2.78)	30 (20.83)	< 0.001
**COVID-19 specific mental well-being, n (%)**^**b**^			
Discontent^c^	.	108 (75.00)	.
Concerned^d^	.	110 (76.39)	.
Afraid^e^	.	90 (62.50)	.
**Change in mental well-being, n (%)**^**f**^			
Same or better	.	38 (26.39)	.
Worse	.	106 (73.61)	.
**Change in physical activity, n (%)**^**f**^			
Same or increase	.	56 (38.89)	.
Decrease	.	88 (61.11)	.
**Change in time spent sitting, n (%)**^**f**^			
Same or increase	.	115 (91.26)	.
Decrease	.	11 (8.73)	.

*MWB* Mental well-being, *PA* Physical activity, *SB* Sedentary behavior

^a^McNemar test

^b^Parents reporting if mental well-being component was sometimes true or true about their child

^c^Discontent with stay-at-home orders

^d^Concerned about COVID-19

^e^Afraid of self or family getting sick

^f^Perceptions of change based on questions asking parents to report on child behavior and mental well-being prior to and during COVID-19 restrictions

Results from chi-squared and logistic regression analyses are presented in Table 3. Discontentment (*x*^2^ (2)=8.06, *p* = 0.005) and concern (*x*^2^ (2)=7.20, *p* = 0.007) about COVID-19 were significantly associated with perceived changes in MWB. Among PA behaviors surveyed, perceived changes in PA (*x*^2^ (2)=4.10, *p* = 0.043), perceived changes in organized sports (*x*^2^ (2)=5.61, *p* = 0.018), and perceived changes in outside play (*x*^2^ (2)=6.35, *p* = 0.012), were significantly associated with perceived changes in MWB. Among SB, only a perceived change in time spent sitting (*x*^2^ (2)=4.65, *p* = 0.031) was statistically significant.

**Table 3:** Analyses of COVID-19 concern, PA, and SB with perceived change in MWB during COVID-19 (*n* = 144)

	Correlation Coefficient (R)	Unadjusted models			Models adjusted for age		
		B (SE)	OR	95% CI	B (SE)	OR	95% CI
**COVID-19 concern**^**a**^							
Discontent	8.06**	− 1.14 (0.41)	0.32**	0.14, 0.72	−1.14 (0.41)	0.32**	0.14, 0.72
Concerned	7.20**	−1.09 (0.42)	0.34**	0.15, 0.76	−1.11 (0.42)	0.33**	0.15, 0.75
Afraid	3.44	.	.	.		.	.
**PA behaviors**^**b**^							
Decrease in PA	4.10*	−0.77 (0.38)	0.46*	0.22, 0.98	−0.77 (0.38)	0.47*	0.22, 0.99
Decrease in organized sports	5.61*	−1.05 (0.45)	0.35*	0.14, 0.85	−1.07 (0.46)	0.34*	0.14, 0.84
Decrease in outside play	6.35*	−1.27 (0.53)	0.28*	0.10, 0.79	−1.26 (0.53)	0.28*	0.10, 0.80
Decrease in inside play	1.22	.	.	.		.	.
Decrease in family PA	0.81	.	.	.		.	
**SB**^**c**^.							
Same or increase in time sitting	4.65*	−1.32 (0.64)	0.27*	0.08, 0.95	−1.22 (0.65)	0.30	0.08, 1.06
Same or increase in screen time for entertainment	0.00	.	.	.		.	.
Same or increase in screen time for education	1.31	.	.	.		.	.

Statistical Significance at the *P* < 0.05 (*), *p* < 0.01(**) level

*PA* Physical activity, *SB* Sedentary behaviors, *MWB* Mental well-being

MWB, PA, and SB changes are perceived changes based on questions asking parents to report on child MWB, PA, and SB prior to and during COVID-19 restrictions

^a^Reference group for COVID-19 concern (not true)

^b^Reference group for PA behaviors (same or increase)

^c^Reference group for SB (decrease)

Logistic regressions were conducted for all statistically significant correlations. Models were adjusted for age and gender to control for potential confounding of these demographic factors. Overall, outcomes did not change significantly after adjusting for age and gender. However, there was evidence of confounding by age for change in sitting. Therefore, results are presented for both unadjusted and age-adjusted models (Table 3). We found children whose parents reported them to be discontent with stay-at-home orders were 68% less likely to have the same or better MWB during COVID-19 restrictions compared to children who were content with stay-at-home orders (OR = 0.32, 95CI 0.14–0.72). Similarly, if a child was concerned about COVID-19, they were 67% less likely to have the same or better MWB during COVID-19 restrictions compared to a child who was not concerned about COVID-19 (OR = 0.33, 95CI 0.15–0.75). In terms of PA behaviors, if a child’s perceived PA decreased following COVID-19 restrictions, they were 53% less likely to have the same or better MWB than a child whose perceived PA stayed the same or increased (OR = 0.47, 95CI 0.22–0.99). Similarly, if a parent perceived a child’s participation in organized sports decreased, they were 66% less likely to have the same or better MWB during COVID-19 restrictions than a child whose perceived participation in organized sports stayed the same or increased (OR = 0.34, 95CI 0.14–0.84). A child whose parents perceived a decrease in playing outside during COVID-19 restrictions were 72% less likely to have the same of better MWB during COVID-19 restrictions than a child whose perceived time playing outside stayed the same or increased (OR = 0.28, 95CI 0.10–0.80). Regarding SB, time spent sitting was no longer significant after adjusting for age (OR = 0.30, 95CI 0.08–1.06).

### Qualitative results

Themes from qualitative interviews provided context to how COVID-19 restrictions impacted children’s MWB and elucidated protective factors which minimized the impact of COVID-19 (Table 4). A prominent theme revolved around the adjustment to stay-at-home orders and COVID-19 restrictions. Many parents reported their children feeling frustrated by COVID-19 restrictions and having difficulty adjusting to online school and a lack of peer interactions. Parents reported it was difficult for children to adapt to this new routine and often complained of missing friends and school. Some parents reported children being emotionally up and down or regressing due to a lack of social interaction they would have had at school. When discussing the upcoming school year, many parents reported their children were disappointed about the prospect of not returning to in-person school. Another prominent theme developed around child understanding and concern about COVID-19. Parents’ discussions about COVID-19 varied with a child’s age. Most parents reported their child had some level of understanding and acknowledgement that COVID-19 causes people to be sick and can be passed through contact with other people. Children seemed to take precautions seriously. Many children, especially at the beginning of the outbreak expressed worry and fear around their family getting sick. A final theme emerged around ways of protecting against the MWB effects of COVID-19 restrictions. Parents felt children benefited from being outside and staying active. While some parents indicated limitations in PA due to closure of parks and organized sports such as dance and soccer, many noted playing outside in a yard, riding bikes, and taking walks improved child MWB. Additionally, many parents reported the use of technology for socializing to be helpful in minimizing some of the loneliness due to COVID-19 restrictions. Many children used video calls, multiplayer games, and online chats to interact with friends remotely.

**Table 4:** Child mental well-being themes during COVID-19 restrictions

Theme	Sub-Themes	Definition	Examples
Adjusting to COVID-19 restrictions	Limitations -no in-person school -change in routine Boredom/Monotony -stuck at home -online school Lack of Socializing -no in-person interaction -no organized sports	The impact of COVID-19 and SAH orders on daily life. This includes in-person school closures, park closures, organized sports closures, online learning, staying at home, and reduced in-person contact.	“I don’t understand why we can’t do these things … I don’t understand why I can’t go to the playground” “He’s like, ‘I thought we were going to get to go back to normal at some point.’ And he was really excited about starting kindergarten and he was like, ‘I don’t get to do that?’” “I think the impact is coming from just the extensive amount of time that we have all spent in the same house” “She’s definitely been very up and down. She really missed seeing friends, missed seeing extended family, missed going places. She really missed school, that was really hard for her …” “He misses his friends a lot. When we drive by the school, he gets pretty emotional …”
COVID-19 concern	Understanding -transmission -symptoms -severity Health concerns -family and friends -self Prevention -hand washing -staying at home -limiting contact with people	What children understand about COVID-19, the reasons for COVID-19 restrictions, mechanisms for transmission, symptoms, and severity. How concerned they are about themselves, family, and friends getting sick and the importance of prevention.	“He talks about a matter-of-factly. ‘Well, we can’t do X, Y, and Z because of the germs. Are the germs going to be over yet so that we can do X, Y, and Z? or we have to wash your hands because of the germs are outside’” “He asks lots of questions about it and on occasion he will express concern like, “Will I get sick? Are you sick? Are you going to get sick?” “Her concern would just be if her or a family member would become ill, what would become of it, that’s her fear.” “She is concerned about it when we started kind of seeing like grandparents again, she was nervous to hug them or touch them or get too close to them … So it definitely made her more cautious.”
What helps with mental well-being during COVID-19	Activity -getting outside -physical activity Social Technology -facetime -multi-player games -online chats	What aspects of daily living helped mitigate the mental well-being effects of COVID-19 restrictions.	“Staying active is key during this time.” “As the weather got better and we could be outside doing more, I think some of that was alleviated just because he didn’t feel so confined.” “They do a lot of FaceTime and some of those apps like House Party and things like that so they can actually see each other and talk and a group of them being together.” “I did notice that once the learning plan started up and they were having the Zoom calls with their teachers and she could talk to her friends … her mood greatly improved. And she hasn’t been as affected since then.”

## Discussion

The findings of this study suggest COVID-19 restrictions in the spring and early summer of 2020 may have impacted child MWB, PA, and SB. Through a mixed-methods approach, we were able to identify potential mechanisms through which these restrictions could impact children and potential ways of mitigating these impacts. While our results are based on parent perceptions around their child’s behavior and well-being during COVID-19 restrictions, this research presents novel findings during an unprecedented time, and provides foundations for further understanding about the role of COVID-19 restrictions on child MWB, PA, and SB.

Parents perceived MWB to decrease for all MWB items in the adapted child Mood and Feelings Questionnaire and 74% of parents reported worse child MWB with COVID-19 restrictions. These findings support previous research highlighting the vulnerability of child MWB to the COVID-19 pandemic [33–35]. Qualitative analysis conducted in this study suggest in-person school closures and changes to daily life (e.g. limited contact with friends and family) due to COVID-19 restrictions, contributed to worsening child MWB. Transitions to remote learning not only posed educational challenges, but according to our findings increased feelings of loneliness and unhappiness for children who missed school activities, such as school sports and socializing with friends. Participants also reported increased worry for children, noting they felt the restrictions of not seeing grandparents, attending in-person school, or being able to use the local playground, and either drew scary conclusions about COVID-19 or were unsure what to think about the COVID-19 pandemic. These findings are particularly important given many schools are continuing to be remote throughout the fall of 2020 and spring of 2021, and daily life remains altered from life prior to the COVID-19 pandemic [8–10, 12].

Another important finding of our study was the association between COVID-19 restrictions and reduced PA. This finding adds to previous research which has found a similar association among adults and children alike [36–40]. Reduced PA is problematic not only because of the importance of PA in preventing obesity and chronic disease later in life, but also because of the associations we found between PA with perceived changes in MWB [22]. In particular, the amount of time playing outside was perceived by parents to have the greatest impact on changes in MWB during COVID-19 restrictions. During interviews parents reported their child missed activities like playing on the local playground and spending time outside with friends. Parents in our study overwhelmingly felt their child’s mood and behavior improved after spending time outside playing in the yard, riding bikes, or taking walks with family. This finding supports previous research suggesting outside play and connection with nature have benefits for mental health and concentration [41–44].

We did not find associations between time spent sitting and changes in MWB after adjusting for age. However, many parents we interviewed, noted that not having the structure of in-person school, along with the added screen time of remote learning, increased SB and in particular time spent sitting, which was often reported to be difficult for children. Parents felt their child’s mood and behavior were negatively impacted, citing how their child often complained of boredom and difficulty concentrating in the remote learning environment. The differences between our quantitative and qualitative findings regarding SB and child MWB, highlight the need for more research into the potential impact of age on child experiences during COVID-19.

With ongoing restrictions, efforts should be directed toward increasing socialization remotely, improving communication with children about COVID-19, and finding ways to increase PA and reduce SB during the COVID-19 pandemic. One way many parents identified as an opportunity to increase socialization was through the use of technology. Many parents felt using technology to socialize through multi-player games, video calls, and social media alleviated some of the loneliness their children were feeling due to COVID-19 restrictions. These findings align with research highlighting how technology can be used to maintain social connection during the COVID-19 pandemic [45, 46]. While technology may help improve social connection remotely, it can also lead to increased SB and adverse health outcomes such as increased risk of obesity and poor mental health [47–49]. As such, it is important to consider the balance between the need for socializing to protect against loneliness and the imperative to reduce time spent sitting in front of a screen. Some potential alternatives may be more active socializing through technology [50–53]. Examples of technology use for being active and social could include a dance class over video call or remote yoga with friends. As COVID-19 restrictions continue, schools may find it beneficial to use technology to create virtual physical education classes, where students can be active while interacting. School interventions and policies should focus on incorporating technology into the remote learning space that promotes PA and supports social connection amongst students.

In discussing child worry and concern about COVID-19, many parents in this sample reported children often worried less after having conversations about restrictions being in place to prevent the transmission of COVID-19 and ways family and friends were protecting themselves from becoming sick. Having these conversations in age-appropriate ways may help alleviate worry and fear some children might experience due to the COVID-19 pandemic and associated restrictions. The Centers for Disease Control and Prevention (CDC) have disseminated guidelines on the importance of communicating with children about COVID-19 and how to speak with children of different ages [54]. Other organizations have also supported age appropriate discussion with children, being open to a child’s questions, and keeping lines of communication open [55, 56]. Further, schools can act as important sources of information for parents on how to have age appropriate conversations. Collaboration between schools and parents of elementary school aged children should be ongoing to facilitate communication about COVID-19 and how to talk with children about the ongoing pandemic.

Our findings on PA and SB suggest a need for interventions and policies to promote PA and reduce SB during the COVID-19 pandemic and associated restrictions. Outside play was perceived to have the greatest impact on changes in child MWB, which may provide opportunities to promote PA in warmer months but suggests PA may decrease in winter months. While weather is conducive to outdoor activity, family walks or bike rides outside and making time each day for playing in the yard may be options. Additionally, outdoor activity can provide safer conditions for socializing aligning with recommendations for preventing the spread of COVID-19 [57]. If weather is not conducive to outdoor play, children should be encouraged to spend 10–15 minutes being active indoors through activities such as stretching, sit-ups or jumping jacks for every hour spent sitting in front of a screen [58]. Activity breaks will aid in maintaining levels of PA when being active outside due to weather is not an option. Schools should incorporate these short exercises into the remote learning setting. Class time PA breaks have been shown to be effective for increasing PA and preventing obesity in the elementary school population [59, 60]. Adapting PA breaks to remote learning may help children maintain PA levels during COVID-19 restrictions and through colder weather when outside play is not as feasible.

There are a few limitations of this study. First, the convenience sample is small and homogeneous, limiting some opportunities for analysis and generalizability of findings to other populations. While the survey overall had 245 respondents, we were only able to conduct analysis on the 144 respondents who completed the adapted child Mood and Feelings questionnaire. Second, this study was cross-sectional in nature. As such, we can only assess associations between child MWB, PA, and SB during COVID-19 restrictions and cannot make causal inferences. Third, since we only collected data during the months of May through July, our study cannot assess how a longer time spent at home, or how time of year and weather might impact child MWB, PA, and SB. Finally, measures of change in MWB, PA, and SB relied on parent-report and were subject to recall bias. We also did not collect data on weekly minutes of moderate and vigorous PA, limiting our ability to use U.S. physical activity guidelines to assess perceived changes in child PA. Although limited by sample size and parent-report, this study used a mixed methods approach, which provided context and corroboration for quantitative findings from the online survey. Findings of this study also help contribute to new research in the area of COVID-19 and specifically for how COVID-19 restrictions impact children.

## Conclusion

This study captured perceived changes in MWB, PA and SB at the start of the COVID-19 pandemic through a mixed-methods approach. Based on our findings, COVID-19 had a significant impact on child MWB, with children experiencing worse MWB due to COVID-19 restrictions. According to parents in this study, child PA behaviors decreased, SB increased with COVID-19 restrictions and maintaining or increasing child PA levels and activities acted as a protective factor for child MWB. Our results indicate monitoring child MWB, PA and SB during this ongoing pandemic is critical to identify ways to intervene and prevent potential long-term consequences of poor MWB, decreased PA, and increased SB. Schools and parents should look towards communicating with children about the COVID-19 pandemic to reduce fear and worry, increasing outdoor PA as weather permits, and incorporating short activities to break up SB and maintain PA levels.

As the COVID-19 pandemic and associated restrictions continue, ongoing research will be needed to determine effective interventions for child MWB and ways of keeping children active and healthy during these unprecedented times. More information is needed on how COVID-19 restrictions may differentially impact children by race and ethnicity, socioeconomic status, and urbanicity. Additionally, with COVID-19 restrictions ongoing, it will be critical to determine the role of seasonality and duration of COVID-19 restrictions on child MWB, PA, and SB. While this study contributes to an initial understanding of how COVID-19 restrictions may impact child MWB, PA, and SB, more research will be needed to more fully understand both immediate and long-term outcomes for children.

## Supplementary Information

**Additional file 1: Supplement 1**. Online Questionnaire

## Data Availability

The datasets generated and/or analyzed during the current study are available from the corresponding author on reasonable request.

## Abbreviations

COVID-19: Coronavirus disease 2019
MWB: Mental well-being
PA: Physical activity
SB: Sedentary behavior
