# Supplementary material for: Associations of physical activity and sedentary behaviors with child mental well-being during the COVID-19 pandemic
Source: BMC Public Health. 2021 Sep 28;21:1770. doi: 10.1186/s12889-021-11805-6 (PMC8478004; doi:10.1186/s12889-021-11805-6)
Supplement: Supplementary file 1 — Additional file 1: Supplement 1. Online Questionnaire [file 12889_2021_11805_MOESM1_ESM.docx]

Changes in children's activity and screen time during COVID-19 stay-at-home

Start of Block: Project Information

Q7 We appreciate your willingness to complete this survey. This is a unique time for all of us. From this survey, we hope to understand how COVID stay-at-home policies have influenced your child's physical activity and screen time. We are surveying parents with the goal of identifying ways we can help communities lead healthy lifestyles during this time and beyond. 
 
The survey is anonymous, so you will not be asked for any identifying information. If you have any questions about the research study itself, please contact: Amy Eyler at (314) 935-0129. If you have questions, concerns, or complaints about your rights as a research participant, please contact the Human Research Protection Office at 660 South Euclid Avenue, Campus Box 8089, St. Louis, MO  63110, 1-(800)-438-0445 or email [hrpo@wustl.edu](mailto:hrpo@wustl.edu). General information about being a research participant can be found on the Human Research Protection Office web site, <http://hrpo.wustl.edu/.>  To offer input about your experiences as a research participant or to speak to someone other than the research staff, call the Human Research Protection Office at the number above.

- I would like to take the survey
- No thanks. I don't want to take the survey.

| Page Break |  |
| --- | --- |

End of Block: Project Information

Start of Block: Introduction and Child Demographics

Q5 Questions in this survey should be answered about you and your child who is between kindergarten and 5th grade level. If you have more than one child who falls in this age range, **please select one child** to answer questions about for the remainder of the survey.

Q1 What is your child’s age in years?

________________________________________________________________

Q2  What is your child's gender?

- Male
- Female
- Non-binary or third gender
- Prefer to self-describe: ________________________________________________

| Page Break |  |
| --- | --- |

End of Block: Introduction and Child Demographics

Start of Block: Values and importance

Q3 The questions in this section are about you, and what you think and feel. Answer each to the best of your ability.

| 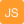 |
| --- |

Q4 How much do you agree or disagree with each of the following statements? Mark the response which best reflects how much you agree or disagree.

|  | Strongly disagree | Disagree | Somewhat disagree | Neither agree nor disagree | Somewhat agree | Agree | Strongly agree |
| --- | --- | --- | --- | --- | --- | --- | --- |
| Participating in regular physical activity is important to me. |  |  |  |  |  |  |  |
| Having my child be active regularly is important to me. |  |  |  |  |  |  |  |
| I find my participation in physical activity valuable. |  |  |  |  |  |  |  |
| I find my child’s participation in physical activity valuable. |  |  |  |  |  |  |  |

Q6 Which of the following do you find valuable about your child’s participation in physical activity during the COVID-19 stay-at-home? Please rank these from most important (1) to least important (5). You may leave items that you do not find valuable blank.

______ Energy release (e.g., “get your sillies out”)

______ Sleep quality

______ Physical health benefits (e.g., growth and development)

______ Mental health (e.g., regulating emotions)

______ Other: please describe

| Page Break |  |
| --- | --- |

End of Block: Values and importance

Start of Block: Child PA and screen time behaviors

Q9 Please respond to each of the following questions about your child's physical activity and screen time behaviors to the best of your ability.

Q8 What does your child usually do when s/he has a choice about how to spend their free time?

- almost always chooses sedentary activities, such as watching TV, playing video games, or reading
- usually chooses sedentary activities, such as watching TV, playing video games, or reading
- just as likely to choose physically active play as inactive recreation
- usually chooses physically active play
- almost always chooses physically active play

Q10 Overall, **during** the COVID-19 stay-at-home orders, do you feel your child’s physical activity has:

- Decreased
- Stayed the same
- Increased

Q11 On an average day, **during** the COVID-19 stay-at-home period, how many minutes does your child spend doing each of the following:

|  | During the stay-at-home period | Is this different than before stay-at-home? | | |
| --- | --- | --- | --- | --- |
|  | (mintues per day) | Less | About the same | More |
| using screens (TV, tablets, computers, smartphones, videogames) alone for entertainment |  |  |  |  |
| watching TV/movies or playing video games as a family for entertainment |  |  |  |  |
| using screens for educational purposes |  |  |  |  |
| sitting or lying down (do not count sleep) |  |  |  |  |
| playing inside |  |  |  |  |
| playing outside |  |  |  |  |
| participating in sports or organized activities |  |  |  |  |
| playing outdoors as a form of family recreation |  |  |  |  |
| using physical activity or sports as a form of family recreation, indoors or outdoors (e.g., going on a bike ride together, hiking, walking) |  |  |  |  |

Q12 How often does your child move around, jump, or dance while watching TV or other screens?

- Never
- Rarely
- Occasionally
- Sometimes
- Often
- Very often/always

| Page Break |  |
| --- | --- |

End of Block: Child PA and screen time behaviors

Start of Block: Barriers to promoting healthy PA and ST behaviors

Q13 During stay-at-home my child's physical activity has been limited by... (select all that apply)

- My child's lack of interest or motivation
- Lack of adult supervision
- My own lack of motivation and interest
- My busy schedule
- Other adults (for example, a co-parent) in my child's life
- Lack of support from my family, spouse, or friends
- Lack of other children to play with
- Size or layout of my indoor spaces
- Size or lack of a yard at my household
- Safety of my neighborhood
- Lack of play spaces available or open in my neighborhood

| 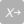 |
| --- |

Q14 Please rank the answers that you selected from the previous question in order of the most (top) to least (bottom) impact on your child's physical activity right now.

______ My child's lack of interest or motivation

______ Lack of adult supervision

______ My own lack of motivation and interest

______ My busy schedule

______ Other adults (for example, a co-parent) in my child's life

______ Lack of support from my family, spouse, or friends

______ Lack of other children to play with

______ Size or layout of my indoor spaces

______ Size or lack of a yard at my household

______ Safety of my neighborhood

______ Lack of play spaces available or open in my neighborhood

Q15 During stay-at-home, how often do things keep your child from being physically active regularly?

- Never
- Rarely
- Occasionally
- Sometimes
- Often
- Very often or always

| Page Break |  |
| --- | --- |

End of Block: Barriers to promoting healthy PA and ST behaviors

Start of Block: Parent influence over child PA and ST

Q66 The questions in this section are about your thoughts and opinions of several topics related to your child. Answer each to the best of your ability.

Q17 How much do you agree or disagree with each of the following statements? Mark the response which best reflects how much you agree or disagree.

Q16 I have influence over how much physical activity my child gets

- Strongly disagree
- Disagree
- Somewhat disagree
- Neither agree nor disagree
- Somewhat agree
- Agree
- Strongly agree

Q18 I have influence over how much screen time (e.g., television, video games, computers, tablets) my child has.

- Strongly disagree
- Disagree
- Somewhat disagree
- Neither agree nor disagree
- Somewhat agree
- Agree
- Strongly agree

Q19 How much do you use your own behavior to encourage your child to be physically active?

- I don't use my own behavior to encourage my child to be active.
- I rarely use my own behavior to encourage my child to be active.
- I often use my own behavior to encourage my child to be active.
- I constantly use my own behavior to encourage my child to be active.

Q20 Has this changed since the COVID-19 stay-at-home began?

- No
- Yes

Q21 How has your influence changed?

- I am not able to use my own behavior as much to encourage my child to be active.
- I am able to use my own behavior more to encourage my child to be active.

Q24
On the scale provided, please indicate how often you do each of the following.

|  | During the COVID-19 stay-at-home, how often do you or another adult in the household… | | | | | | Has this changed since stay-at-home began? How so? | | |
| --- | --- | --- | --- | --- | --- | --- | --- | --- | --- |
|  | Never | Rarely | Occasionally | Sometimes | Often | Very often or always | Less often now | About the same | More often now |
| Turn on the TV, a video/movie, or a computer/tablet for your child when the weather is bad (for example, raining, too hot, too cold)? |  |  |  |  |  |  |  |  |  |
| Get out a toy or piece of equipment without being asked that will be used by your child during moderate or vigorous physical activity? |  |  |  |  |  |  |  |  |  |
| Start a physically active game with your child? |  |  |  |  |  |  |  |  |  |
| Send your child outside to play? |  |  |  |  |  |  |  |  |  |
| Turn on the TV, a video/movie, or a computer/tablet for your child so you can get things done around the house (e.g., laundry, dishes, etc.)? |  |  |  |  |  |  |  |  |  |
| Turn on the TV, a video/movie, or a computer/tablet for your child so you can do work at home? |  |  |  |  |  |  |  |  |  |
| Say things to encourage your child to physical activities? |  |  |  |  |  |  |  |  |  |
| Say things to encourage your child to be less active (e.g., stop running) |  |  |  |  |  |  |  |  |  |
| Say things to encourage your child to spend less time watching TV/movies or playing video games or on a computer? |  |  |  |  |  |  |  |  |  |

Q25
On the scale provided, please indicate how often you or another adult in your household talk with
your child about these issues.

|  | How often do you or another adult in the household currently… | | | | | | Has this changed since stay-at-home began? How so? | | |
| --- | --- | --- | --- | --- | --- | --- | --- | --- | --- |
|  | Never | Rarely | Occasionally | Sometimes | Often | Very often or always | Less often now | About the same | More often now |
| discuss with your child how being physically active is good for their health? |  |  |  |  |  |  |  |  |  |
| discuss with your child how sedentary habits can be unhealthy? |  |  |  |  |  |  |  |  |  |
| discuss with your child how watching too much TV can be unhealthy? |  |  |  |  |  |  |  |  |  |
| discuss with your child how being physically active builds strong muscles? |  |  |  |  |  |  |  |  |  |

| Page Break |  |
| --- | --- |

End of Block: Parent influence over child PA and ST

Start of Block: Rules, limits, and negotiations about child PA and ST

Q26 On the scale provided please indicate how much you agree or disagree with each statement.

|  | During the COVID-19 stay-at-home, how much do you agree or disagree with each of the following? | | | | | | | Are these rules enforced differently now compared to before stay-in-place began? | | |
| --- | --- | --- | --- | --- | --- | --- | --- | --- | --- | --- |
|  | Strongly disagree | Disagree | Somewhat disagree | Somewhat agree | Agree | Strongly agree | Not applicable | Enforced less now | About the same | Enforced more now |
| My child can only play in the yard where I can see him/her from inside. |  |  |  |  |  |  |  |  |  |  |
| My child can only play in the yard when I or another adult can be outside with him/her. |  |  |  |  |  |  |  |  |  |  |
| My child can only play outside our yard in the neighborhood when I can see him/her. |  |  |  |  |  |  |  |  |  |  |
| My child can only play outside our yard in the neighborhood when I or another adult is with him/her. |  |  |  |  |  |  |  |  |  |  |
| My child can only play outside our yard in the neighborhood with an older sibling is with him/her. |  |  |  |  |  |  |  |  |  |  |

Q27 During COVID-19 stay-at-home, do you limit the amount of screen time your child has during the week (Monday through Friday)?

- No
- Yes

Q28 What are the weekday limits for screen time?

________________________________________________________________

Q29 Has this changed since stay-at-home began?

- Less screen time allowed now
- About the same
- More screen time allowed now

Q30 During COVID-19 stay-at-home, do you limit the amount of screen time your child has during the weekend (Saturday and Sunday)?

- No
- Yes

Q31 What are the weekend limits for screen time?

________________________________________________________________

Q32 Has this changed since stay-at-home began?

- Less screen time allowed now
- About the same
- More screen time allowed now

Q33 How much do you agree or disagree with the following statement: It is hard to limit the amount of screen time my child has.

- Strongly disagree
- Disagree
- Somewhat disagree
- Neither agree nor disagree
- Somewhat agree
- Agree
- Strongly agree

Q34 Please explain why:

________________________________________________________________

Q36 On the scale provided, please indicate how often you do each of the following.

|  | During COVID-19 stay-at-home, how often do you... | | | | | | How has this changed since before stay-at-home began? | | |
| --- | --- | --- | --- | --- | --- | --- | --- | --- | --- |
|  | Never | Rarely | Occasionally | Sometimes | Often | Very often or always | Happening less often now | Happening about the same | Happening more often now |
| offer TV, video, or movie time as a reward for good behavior? |  |  |  |  |  |  |  |  |  |
| take away screen time as a punishment for bad behavior? |  |  |  |  |  |  |  |  |  |
| use screen time to get your child to do something or to control your child’s behavior (example: You can’t watch TV until you clean up your room)? |  |  |  |  |  |  |  |  |  |

Q37 When my child is bored, it helps to turn on the TV or a computer/tablet.

- Strongly disagree
- Disagree
- Somewhat disagree
- Neither agree nor disagree
- Somewhat agree
- Agree
- Strongly agree

Q38 What is one thing that has been positive during this time for your child’s physical activity?

________________________________________________________________

Q67 Do you think this positive change will continue after COVID-19 stay-at-home ends?

- Yes
- No
- Maybe

| Page Break |  |
| --- | --- |

End of Block: Rules, limits, and negotiations about child PA and ST

Start of Block: School resources

Q39 What school does your child attend?

________________________________________________________________

Q40 When did your child’s school close for COVID-19 stay-at-home?

________________________________________________________________

Q41 The next few questions ask about the work assigned by school for your child during shelter at home.

Q42 How much time is required on an average day?

________________________________________________________________

Q43 How much is live (e.g., on Zoom)?

- A great deal
- A lot
- A moderate amount
- A little
- None at all

Q44 How much do you/spouse/partner have to supervise?

- A great deal
- A lot
- A moderate amount
- A little
- None at all

Q45 Has your child’s school kept a requirement for physical education while children are not at school?

- No
- Yes

Q46 Please describe the requirement for physical education during stay-at-home

________________________________________________________________

Q47 What supports for physical activity has your child’s school distributed or promoted since closing for the COVID-19 stay-at-home? (select all that apply)

- Information about the importance of healthy physical activity and screen time behaviors
- Resources to be physically active at home
- Resources to manage appropriate screen time use
- Physical activity equipment (e.g., balls, jump ropes, rhythm scarves, etc)
- Other: please describe ________________________________________________
- My child’s school has not offered any of these things.

| Page Break |  |
| --- | --- |

End of Block: School resources

Start of Block: Child mental well-being

Q68 Please answer whether the below statements are true, not true, or sometimes true about your child before and during stay-at-home orders.

Q69 Click to write the question text

|  | Before stay-at-home orders began, my child... | | | Since stay-at-home orders began, my child... | | |
| --- | --- | --- | --- | --- | --- | --- |
|  | Not true | Sometimes true | True | Not true | Sometimes true | True |
| felt miserable or unhappy |  |  |  |  |  |  |
| didn’t enjoy anything at all |  |  |  |  |  |  |
| felt so tired that she just sat around and did nothing |  |  |  |  |  |  |
| was very restless |  |  |  |  |  |  |
| found it hard to think properly and concentrate |  |  |  |  |  |  |
| felt lonely |  |  |  |  |  |  |
| felt irritable or angry |  |  |  |  |  |  |
| worried a lot |  |  |  |  |  |  |
| had trouble sleeping |  |  |  |  |  |  |

Q70 Since stay-at-home orders began, my child...

|  | Not true | Sometimes true | True |
| --- | --- | --- | --- |
| has not been content with stay-at-home orders |  |  |  |
| was afraid of self/family/friends getting sick |  |  |  |
| was concerned about Covid |  |  |  |
| talked/asked questions about Covid |  |  |  |

| Page Break |  |
| --- | --- |

End of Block: Child mental well-being

Start of Block: Parent and Household Demographics

Q49 Please tell us a little bit more about yourself and your household.

| 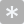 |
| --- |

Q50 How old are you?

________________________________________________________________

Q51 What is your gender?

- Male
- Female
- Non-binary or third gender
- Prefer to self-describe: ________________________________________________

Q52 What is your race? (select all that apply)

- American Indian or Alaska Native
- Asian
- Black or African American
- Native Hawaiian and Pacific Islander
- White
- Other: please describe ________________________________________________

Q53 Would you describe yourself as Hispanic, Latino, or Spanish origin?

- No
- Yes

Q54 What is your current marital status?

- Married or living with a partner
- Single or never married
- Divorced or separated
- Widowed

Q55 What zip code(s) do you live in?

________________________________________________________________

Q56 Are you currently employed (part-time or full-time)?

- No
- Yes

Q57 Are you working mostly from inside or outside your household during COVID-19 stay-at-home?

- Only at home
- Both at home and outside of my home
- Only outside of my home

Q58 Do you share parenting responsibilities for your child with someone who lives outside of your household?

- No
- Yes

Q59 Please describe who you share parenting responsibilities with:

________________________________________________________________

Q60 Does your child regularly live in another household for part of each week or month? This may include things like a joint custody agreement with child's father/mother.

- No
- Yes

Q61 How many days a week does your child spend at your household?

________________________________________________________________

Q62 How many people 18 years of age or older currently live in your home?

________________________________________________________________

Q63 How many people under 18 years of age, including the child you answered these questions about, currently live in your home?

________________________________________________________________

| Page Break |  |
| --- | --- |

End of Block: Parent and Household Demographics

Start of Block: End of survey

Q64 Thank you for taking the time to complete this survey. We value the opportunity to learn about you and your child's experiences with COVID stay-at-home orders. 

 If you have any questions, please contact Amy Eyler at (314) 935-0129.

End of Block: End of survey
